# 4′-Formyl­biphenyl-4-yl acetate

**DOI:** 10.1107/S1600536810001534

**Published:** 2010-01-20

**Authors:** Sheng-guang Xu, Jun-shen Liu, Chun-hua Wang, Chun-nuan Ji, Gang Liu

**Affiliations:** a School of Chemistry & Materials Science, Ludong University, Yantai of Shandong, People’s Republic of China

## Abstract

In the title compound, C_15_H_12_O_3_, the dihedral angle between the six-membered rings is 30.39 (1)°. The crystal packing is stabilized by inter­molecular C—H⋯O hydrogen bonds.

## Related literature

For further synthetic details, see: Chakraborti & Gulhane (2003 ▶); Chamontin *et al.* (1999 ▶); Steglich & Höfle (1969 ▶).
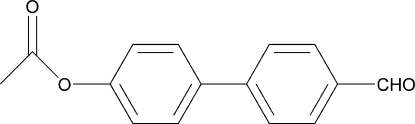


## Experimental

### 

#### Crystal data

C_15_H_12_O_3_
*M* *_r_* = 240.25
Monoclinic, 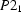

*a* = 9.250 (6) Å
*b* = 7.499 (4) Å
*c* = 9.596 (6) Å
β = 113.695 (10)°
*V* = 609.5 (6) Å^3^
*Z* = 2
Mo *K*α radiation
μ = 0.09 mm^−1^
*T* = 298 K
0.18 × 0.15 × 0.10 mm

#### Data collection

Bruker SMART APEXII CCD-detector diffractometer
Absorption correction: multi-scan (*SADABS*; Sheldrick, 2004 ▶) *T*_min_ = 0.984, *T*_max_ = 0.991
3288 measured reflections
1290 independent reflections
1077 reflections with *I* > 2σ(*I*)
*R*_int_ = 0.019

#### Refinement

*R*[*F* ^2^ > 2σ(*F* ^2^)] = 0.034
*wR*(*F* ^2^) = 0.091
*S* = 1.00
1290 reflections
164 parameters
1 restraint
H-atom parameters constrained
Δρ_max_ = 0.09 e Å^−3^
Δρ_min_ = −0.12 e Å^−3^

### 

Data collection: *APEX2* (Bruker, 2004 ▶); cell refinement: *SAINT* (Bruker, 2004 ▶); data reduction: *SAINT*; program(s) used to solve structure: *SHELXS97* (Sheldrick, 2008 ▶); program(s) used to refine structure: *SHELXL97* (Sheldrick, 2008 ▶); molecular graphics: *XP* in *SHELXTL* (Sheldrick, 2008 ▶); software used to prepare material for publication: *SHELXL97*.

## Supplementary Material

Crystal structure: contains datablocks I, global. DOI: 10.1107/S1600536810001534/bg2320sup1.cif

Structure factors: contains datablocks I. DOI: 10.1107/S1600536810001534/bg2320Isup2.hkl

Additional supplementary materials:  crystallographic information; 3D view; checkCIF report

## Figures and Tables

**Table 1:** Hydrogen-bond geometry (Å, °)

*D*—H⋯*A*	*D*—H	H⋯*A*	*D*⋯*A*	*D*—H⋯*A*
C15—H15*C*⋯O3^i^	0.96	2.41	3.372 (5)	177

Symmetry code: (i) 
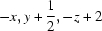
.

**Fig. 1.**
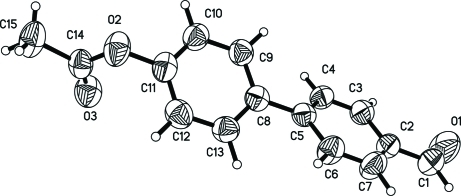
Displacement ellipsoid plot of (I) (50% probability level).

## supplementary crystallographic information

### Comment

4'-formylbiphenyl-4-yl acetate is a derivative of biphenyl that contains two phenyl groups joined by a single covalent bond. However, rotation about the single bond in biphenyl may be sterically hindered and the equilibrium torsional  angles in the variously substituted derivatives may be different. In the  case of unsubstituted biphenyl, the equilibrium torsional angle is 44.4°. However, in the title compound its value is 30.39 (1)°, because conjugated  effects in the molecule are strengthened by π-π conjugation (aldehyde group, oxygen group) as compared with unsubstituted biphenyl, and as result the two phenyl groups tend to be more coplanar.

The molecule of the title compound is illustrated in Fig. 1. The asymmetric unit contains two six-member rings and one acetate group. In the crystal structure there are no classic hydrogen  bonds  but there are non-classical intermolecular C—H···O hydrogen bonds  (viz., C15—H15C···O3[-x,1/2+y,2-z],H···O: 2.41Å, C—H···O: 177°) which help in forming a three dimensional structure.

### Experimental

4'-hydroxy-4-biphenylaldehyde (02) was prepared (Chamontin *et al.* 1999), starting from 4'-bromo-4-biphenol (01) which is commercially available, using the *N*-formylpiperidine as electrophilic agent. After 4'-hydroxy-4-biphenylaldehyde (02) was acetylated with the acetylating agent acetic anhydride, catalyzed by 4-(dimthylamino)pyridine (DMAP), the target material 4'-formylbiphenyl-4-ylacetate (B_3_) was obtained (Chakraborti *et al.* 2003; Steglich *et al.* 1969).

Product, yielding 53%. ^1^H NMR (400MH*_z_*,CDCl_3_, TMS) δ 10.0939 (s, 1H), δ 7.996–7.976 (d, 2H), δ 7.772–7.752(d, 2H), δ 7.688–7. 666 (d, 2H), δ 7.256–7. 235(d, 2H), 2.374 (s,3*H*); ^13^C NMR (CDCl_3_)δ191.79, 169.40, 151.03, 146.28, 137.46, 135.25, 130.29, 128.45, 127.64, 122.19,21.147; ESI-TOF Eaxct Mass for C_15_H_12_O_3 _[*M*+Na]^+^: calcd 263.0679, 264.0713, 265.0738, found 263.0401, 264.0444, 265.0743.

### Refinement

All H atoms were positioned in calculated positions, with C—H distancesof 0.97 Å, and with U*iso*~(H) = 1.2 or 1.5 U*eq*~(C). In the abscense of significan anomalous effects, Friedel pairs were merged, thus giving rise to a poorer reflections to parameters ratio.

### Crystal data

**Table:**

C_15_H_12_O_3_	*F*(000) = 252
*M**_r_* = 240.25	*D*_x_ = 1.309 Mg m^−^^3^
Monoclinic, *P*2_1_	Mo *K*α radiation, λ = 0.71073 Å
Hall symbol: P 2yb	Cell parameters from 1236 reflections
*a* = 9.250 (6) Å	θ = 2.3–23.9°
*b* = 7.499 (4) Å	µ = 0.09 mm^−^^1^
*c* = 9.596 (6) Å	*T* = 298 K
β = 113.695 (10)°	Block, colourless
*V* = 609.5 (6) Å^3^	0.18 × 0.15 × 0.10 mm
*Z* = 2	

### Data collection

**Table:**

Bruker SMART APEX CCD-detector diffractometer	1290 independent reflections
Radiation source: fine-focus sealed tube	1077 reflections with *I* > 2σ(*I*)
graphite	*R*_int_ = 0.019
ω scans	θ_max_ = 26.0°, θ_min_ = 2.3°
Absorption correction: multi-scan (*SADABS*; Sheldrick, 2004)	*h* = −11→11
*T*_min_ = 0.984, *T*_max_ = 0.991	*k* = −7→9
3288 measured reflections	*l* = −8→11

### Refinement

**Table:**

Refinement on *F*^2^	Primary atom site location: structure-invariant direct methods
Least-squares matrix: full	Secondary atom site location: difference Fourier map
*R*[*F*^2^ > 2σ(*F*^2^)] = 0.034	Hydrogen site location: inferred from neighbouring sites
*wR*(*F*^2^) = 0.091	H-atom parameters constrained
*S* = 1.00	*w* = 1/[σ^2^(*F*_o_^2^) + (0.0452*P*)^2^ + 0.0661*P*] where *P* = (*F*_o_^2^ + 2*F*_c_^2^)/3
1290 reflections	(Δ/σ)_max_ = 0.001
164 parameters	Δρ_max_ = 0.09 e Å^−^^3^
1 restraint	Δρ_min_ = −0.12 e Å^−^^3^

### Special details

**Table:**

Geometry. All e.s.d.'s (except the e.s.d. in the dihedral angle between two l.s. planes) are estimated using the full covariance matrix. The cell e.s.d.'s are taken into account individually in the estimation of e.s.d.'s in distances, angles and torsion angles; correlations between e.s.d.'s in cell parameters are only used when they are defined by crystal symmetry. An approximate (isotropic) treatment of cell e.s.d.'s is used for estimating e.s.d.'s involving l.s. planes.
Refinement. Refinement of *F*^2^ against ALL reflections. The weighted *R*-factor *wR* and goodness of fit *S* are based on *F*^2^, conventional *R*-factors *R* are based on *F*, with *F* set to zero for negative *F*^2^. The threshold expression of *F*^2^ > σ(*F*^2^) is used only for calculating *R*-factors(gt) *etc*. and is not relevant to the choice of reflections for refinement. *R*-factors based on *F*^2^ are statistically about twice as large as those based on *F*, and *R*- factors based on ALL data will be even larger.

### Fractional atomic coordinates and isotropic or equivalent isotropic displacement parameters (Å^2^)

**Table:**

	*x*	*y*	*z*	*U*_iso_*/*U*_eq_	
O2	−0.0502 (2)	0.8608 (3)	0.7519 (2)	0.0701 (5)	
C8	0.2140 (3)	0.8713 (3)	0.4862 (3)	0.0477 (5)	
C4	0.2728 (3)	0.7574 (3)	0.2696 (3)	0.0513 (6)	
H4	0.1884	0.6789	0.2457	0.062*	
C5	0.3071 (2)	0.8734 (3)	0.3918 (2)	0.0473 (5)	
C11	0.0414 (3)	0.8599 (3)	0.6654 (3)	0.0560 (6)	
C2	0.4874 (3)	0.8714 (4)	0.2161 (3)	0.0554 (6)	
C9	0.0550 (3)	0.8248 (3)	0.4255 (3)	0.0542 (6)	
H9	0.0052	0.7970	0.3226	0.065*	
C13	0.2828 (3)	0.9145 (3)	0.6404 (3)	0.0557 (6)	
H13	0.3886	0.9477	0.6841	0.067*	
C3	0.3610 (3)	0.7561 (4)	0.1831 (3)	0.0560 (6)	
H3	0.3355	0.6771	0.1019	0.067*	
C10	−0.0305 (3)	0.8190 (4)	0.5149 (3)	0.0580 (6)	
H10	−0.1366	0.7873	0.4725	0.070*	
C6	0.4340 (3)	0.9888 (4)	0.4230 (3)	0.0604 (7)	
H6	0.4593	1.0690	0.5034	0.073*	
C1	0.5827 (3)	0.8732 (5)	0.1248 (4)	0.0741 (8)	
H1	0.6653	0.9546	0.1514	0.089*	
C12	0.1971 (3)	0.9091 (4)	0.7295 (3)	0.0620 (7)	
H12	0.2446	0.9386	0.8321	0.074*	
C14	−0.0346 (3)	0.7197 (4)	0.8450 (3)	0.0550 (6)	
O3	0.0583 (2)	0.6044 (3)	0.8607 (2)	0.0720 (5)	
C7	0.5228 (3)	0.9866 (4)	0.3372 (3)	0.0662 (7)	
H7	0.6080	1.0640	0.3614	0.079*	
C15	−0.1467 (3)	0.7346 (5)	0.9211 (3)	0.0768 (9)	
H15A	−0.1402	0.6290	0.9798	0.115*	
H15B	−0.2523	0.7478	0.8455	0.115*	
H15C	−0.1196	0.8367	0.9871	0.115*	
O1	0.5613 (3)	0.7773 (5)	0.0187 (3)	0.1034 (9)	

### Atomic displacement parameters (Å^2^)

**Table:**

	*U*^11^	*U*^22^	*U*^33^	*U*^12^	*U*^13^	*U*^23^
O2	0.0865 (12)	0.0605 (11)	0.0839 (12)	0.0192 (11)	0.0557 (11)	0.0128 (11)
C8	0.0530 (12)	0.0361 (11)	0.0536 (13)	−0.0002 (11)	0.0210 (10)	0.0025 (12)
C4	0.0482 (12)	0.0508 (14)	0.0534 (13)	−0.0096 (11)	0.0187 (10)	−0.0039 (12)
C5	0.0485 (12)	0.0398 (11)	0.0506 (12)	0.0013 (11)	0.0168 (10)	0.0055 (12)
C11	0.0675 (15)	0.0435 (13)	0.0675 (15)	0.0122 (14)	0.0380 (13)	0.0117 (13)
C2	0.0494 (12)	0.0603 (15)	0.0592 (14)	0.0015 (13)	0.0244 (11)	0.0081 (14)
C9	0.0576 (13)	0.0523 (15)	0.0524 (13)	−0.0038 (12)	0.0217 (11)	0.0027 (11)
C13	0.0521 (13)	0.0567 (16)	0.0561 (14)	0.0011 (11)	0.0195 (11)	−0.0020 (12)
C3	0.0566 (13)	0.0593 (15)	0.0522 (13)	−0.0017 (12)	0.0218 (11)	−0.0029 (13)
C10	0.0558 (14)	0.0557 (16)	0.0657 (15)	−0.0019 (12)	0.0276 (12)	0.0042 (13)
C6	0.0643 (15)	0.0554 (15)	0.0647 (15)	−0.0155 (13)	0.0291 (13)	−0.0128 (14)
C1	0.0587 (15)	0.091 (2)	0.0785 (18)	0.0027 (18)	0.0339 (14)	0.012 (2)
C12	0.0697 (16)	0.0643 (18)	0.0523 (13)	0.0084 (13)	0.0249 (13)	−0.0027 (13)
C14	0.0675 (15)	0.0529 (15)	0.0496 (13)	−0.0100 (13)	0.0287 (12)	−0.0101 (12)
O3	0.0852 (12)	0.0607 (11)	0.0787 (13)	0.0094 (11)	0.0420 (11)	0.0143 (10)
C7	0.0590 (15)	0.0645 (17)	0.0779 (18)	−0.0182 (15)	0.0304 (14)	−0.0030 (16)
C15	0.094 (2)	0.078 (2)	0.080 (2)	−0.0186 (17)	0.0573 (18)	−0.0183 (17)
O1	0.0920 (15)	0.143 (2)	0.0995 (16)	−0.0075 (17)	0.0638 (14)	−0.0223 (18)

### Geometric parameters (Å, °)

**Table:**

O2—C14	1.355 (3)	C13—C12	1.381 (4)
O2—C11	1.404 (3)	C13—H13	0.9300
C8—C9	1.391 (3)	C3—H3	0.9300
C8—C13	1.394 (3)	C10—H10	0.9300
C8—C5	1.480 (3)	C6—C7	1.378 (4)
C4—C3	1.378 (3)	C6—H6	0.9300
C4—C5	1.391 (3)	C1—O1	1.196 (4)
C4—H4	0.9300	C1—H1	0.9300
C5—C6	1.390 (3)	C12—H12	0.9300
C11—C10	1.360 (4)	C14—O3	1.185 (3)
C11—C12	1.369 (4)	C14—C15	1.493 (3)
C2—C7	1.377 (4)	C7—H7	0.9300
C2—C3	1.385 (4)	C15—H15A	0.9600
C2—C1	1.472 (4)	C15—H15B	0.9600
C9—C10	1.382 (3)	C15—H15C	0.9600
C9—H9	0.9300		
			
C14—O2—C11	117.2 (2)	C11—C10—C9	119.6 (2)
C9—C8—C13	117.3 (2)	C11—C10—H10	120.2
C9—C8—C5	121.6 (2)	C9—C10—H10	120.2
C13—C8—C5	121.1 (2)	C7—C6—C5	121.2 (2)
C3—C4—C5	121.5 (2)	C7—C6—H6	119.4
C3—C4—H4	119.2	C5—C6—H6	119.4
C5—C4—H4	119.2	O1—C1—C2	124.6 (3)
C6—C5—C4	117.3 (2)	O1—C1—H1	117.7
C6—C5—C8	121.5 (2)	C2—C1—H1	117.7
C4—C5—C8	121.2 (2)	C11—C12—C13	119.5 (2)
C10—C11—C12	121.0 (2)	C11—C12—H12	120.3
C10—C11—O2	118.3 (2)	C13—C12—H12	120.3
C12—C11—O2	120.6 (2)	O3—C14—O2	122.2 (2)
C7—C2—C3	118.5 (2)	O3—C14—C15	127.1 (3)
C7—C2—C1	120.0 (3)	O2—C14—C15	110.7 (2)
C3—C2—C1	121.5 (3)	C6—C7—C2	121.0 (2)
C10—C9—C8	121.4 (2)	C6—C7—H7	119.5
C10—C9—H9	119.3	C2—C7—H7	119.5
C8—C9—H9	119.3	C14—C15—H15A	109.5
C12—C13—C8	121.3 (2)	C14—C15—H15B	109.5
C12—C13—H13	119.4	H15A—C15—H15B	109.5
C8—C13—H13	119.4	C14—C15—H15C	109.5
C4—C3—C2	120.5 (2)	H15A—C15—H15C	109.5
C4—C3—H3	119.8	H15B—C15—H15C	109.5
C2—C3—H3	119.8		
			
C3—C4—C5—C6	0.2 (4)	C12—C11—C10—C9	0.8 (4)
C3—C4—C5—C8	−178.6 (2)	O2—C11—C10—C9	177.1 (2)
C9—C8—C5—C6	150.4 (3)	C8—C9—C10—C11	0.2 (4)
C13—C8—C5—C6	−30.2 (3)	C4—C5—C6—C7	−0.7 (4)
C9—C8—C5—C4	−30.8 (3)	C8—C5—C6—C7	178.2 (2)
C13—C8—C5—C4	148.6 (2)	C7—C2—C1—O1	179.4 (3)
C14—O2—C11—C10	103.6 (3)	C3—C2—C1—O1	−0.8 (5)
C14—O2—C11—C12	−80.1 (3)	C10—C11—C12—C13	−1.0 (4)
C13—C8—C9—C10	−1.0 (4)	O2—C11—C12—C13	−177.2 (3)
C5—C8—C9—C10	178.5 (2)	C8—C13—C12—C11	0.2 (4)
C9—C8—C13—C12	0.8 (4)	C11—O2—C14—O3	4.2 (4)
C5—C8—C13—C12	−178.6 (2)	C11—O2—C14—C15	−176.4 (2)
C5—C4—C3—C2	0.0 (4)	C5—C6—C7—C2	0.9 (4)
C7—C2—C3—C4	0.2 (4)	C3—C2—C7—C6	−0.7 (4)
C1—C2—C3—C4	−179.6 (3)	C1—C2—C7—C6	179.2 (3)

### Hydrogen-bond geometry (Å, °)

**Table:**

*D*—H···*A*	*D*—H	H···*A*	*D*···*A*	*D*—H···*A*
C15—H15C···O3^i^	0.96	2.41	3.372 (5)	177

Symmetry codes: (i) −*x*, *y*+1/2, −*z*+2.
